# The impact of breast milk feeding on early brain development in preterm infants in China: An observational study

**DOI:** 10.1371/journal.pone.0272125

**Published:** 2022-11-21

**Authors:** Yao Zhang, Qingqi Deng, Jinhua Wang, Hua Wang, Qiufang Li, Binghua Zhu, Chai Ji, Xinfen Xu, Linda Johnston

**Affiliations:** 1 Department of Midwifery, School of Nursing, Zhejiang Chinese Medical University, Hangzhou, Zhejiang, China; 2 Lawrence S. Bloomberg Faculty of Nursing, University of Toronto, Toronto, Ontario, Canada; 3 Department of Radiology, Children’s Hospital, School of Medicine, Zhejiang University, Hangzhou, Zhejiang, China; 4 Neonatal Intensive Care Unit, Women’s Hospital, School of Medicine, Zhejiang University, Hangzhou, Zhejiang, China; 5 Department of Nursing, The Fourth Affiliated Hospital Zhejiang University School of Medicine, Yiwu, Zhejiang, China; 6 Department of Nursing, Haining Maternal and Child Health Hospital, Haining, Zhejiang, China; Western University, CANADA

## Abstract

**Background:**

The prevailing consensus from large epidemiological studies is that breastfeeding is associated with improved IQ and cognitive functioning in later childhood and adolescence. Current research is exploring the association between breastfeeding and early brain development in preterm infants.

**Objective:**

To explore the differences in brain gray matter between breastmilk-fed and formula-fed preterm infants using structural and functional magnetic resonance imaging.

**Methods:**

A convenience sample of breastmilk-fed preterm infants(n = 34) and formula-fed infants (n = 22) aged approximately 32 weeks. At near term-equivalent age, MR scanning was performed. Gray matter structural and functional differences between the two groups were assessed using MATLAB software for voxel-based morphometry (VBM) and amplitude of low-frequency fluctuation (ALFF) analysis.

**Results:**

Maternal and neonatal demographic characteristics showed no significant difference between the two groups. Breastmilk-fed infants had greater regional gray matter volume on MRI than formula-fed infants in multiple brain regions, including the bilateral frontal lobe (BA11, BA46), right temporal lobe (BA37), and left caudate nucleus, at a statistical threshold of p<0.01 (AlphaSim corrected) with a cluster size of >40 voxels. Compared with formula-fed infants, breastmilk-fed infants showed increased brain activation on fMRI in the right superior temporal gyrus (BA41).

**Conclusion:**

Breastmilk-fed infants had greater regional gray matter development and increased regional gray matter function compared with formula-fed infants at near term-equivalent age, suggesting breastmilk feeding in the early period after birth may have some degree of influence on early brain development in preterm infants.

## Introduction

Worldwide, an estimated 15 million babies are born preterm (before 37 completed weeks of gestation) every year, and this number is rising. Almost 1 million children die each year due to complications of preterm birth [1]. Many survivors face a lifetime of disability, including learning disabilities and visual and hearing problems [2]. More than 60% of preterm births occur in Africa and South Asia. China has the second greatest number of preterm births worldwide [3] with approximately 2 million babies born preterm every year [4]. For these preterm infants, the rate of cerebral palsy is up to 60.6% [5], 55% have learning disabilities when they enter school [6] and 20% need special education [7].

Early breastfeeding is very important for early brain development; it influences brain growth and maturation and exerts subsequent effects on neurodevelopment that persist into childhood and adolescence [8]. The impact of breast milk on brain development in preterm infants has been increasingly appreciated [9]. As the period from 25 weeks gestation to two years of age is the most rapid growth period of human brain development [10], nutrition is especially important for preterm infants in early life. This period is crucial for brain formation and the development of cognitive, behavioral, and socioemotional skills throughout childhood and adulthood [11]. Recent studies has also demonstrated the importance of adequate nutrition for the developing brain [9, 12]. Breast milk contains nutrients that are essential in early infancy, including docosahexaenoic acid (DHA) and long-chain polyunsaturated fatty acids (LC-PUFAs) [13, 14]. Emerging studies suggest that breast milk feeding in infancy is associated with improved brain growth, white matter microstructure, and short- and long-term neurodevelopmental performance in preterm infants [15–18].

Deoni et al.’s study [19] showed that breastfed children from 10 months through four years of age exhibited increased white matter development in the maturing frontal, and associated, brain regions at later stages. Positive relationships were found between white matter microstructures and breastfeeding duration in several brain regions, which are consistent with the observed improvements in cognitive and behavioral performance measures. Also, Blesa M et al.’s study [17] demonstrated predominant breast milk feeding in the weeks after preterm birth is associated with improved structural connectivity of developing networks and greater fractional anisotropy (FA) in major white matter fasciculi. Although there is an increasing number of studies focusing on brain development in the preterm infant at term equivalent age, uncertainties remain about the effect of breast milk on preterm brain development in early life, particularly in Asian populations.

Magnetic resonance imaging (MRI), especially structural imaging, is increasingly recognized as a useful and reliable tool to study brain development. Voxel-based morphometry (VBM) is a whole-brain, voxelwise technique that analyzes local changes (especially gray and white matter changes) in brain tissue [20–22]. Few studies have used VBM to explore gray and white matter changes in preterm infants, and limited reports using VBM to determine gray or white matter changes in breastfed or formula-fed preterm infants in early life have been identified. Structural MRI may be complemented by measures of brain functioning obtained using functional MRI (fMRI). The amplitude of low-frequency fluctuation (ALFF) has been used in several areas of neuroscience and neurological diseases as an index for measuring regional spontaneous neuronal activity in resting-state fMRI [23–25]. Kiviniemi et al. [26] indicated that ALFF may be suggestive of regional spontaneous neuronal activity and an indication of functional changes in brain activity. Therefore, ALFF signals may reflect cerebral physiological states that are useful in evaluating the functional development of the brain.

The purpose of the present study was to assess brain gray matter structure and function using MRI in preterm infants who were fed predominantly breast, or formula, milk during the first year of life. We hypothesized that breast milk promotes greater gray matter development and functioning in preterm infants than formula milk. To test this hypothesis, we compared VBM measures of regional gray matter structure and fMRI measures of brain activation under sedation between breastmilk-fed and formula-fed preterm infants.

## Materials and methods

### Design and setting

This study employed an observational design and was undertaken in the Women’s Hospital, School of Medicine, Zhejiang University, a public maternity hospital in Zhejiang Province of China. This hospital has a bed capacity of 1120, with 105 beds in the NICU. Approximately 6000 preterm infants are delivered in this hospital annually.

### Participants

Preterm infants born in the hospital were recruited between January and December, 2016. The selection criteria were as follows: healthy birth between 30 and 34 weeks of gestation; APGAR score of at least 7 at 5 minutes after birth; no requirement for emergency interventions after birth; no congenital disease or multiple congenital anomaly syndromes on fetal ultrasound; and no reported history of neurological events or disorders (e.g head trauma, ischemia, epilepsy).

Exclusion criteria included: family history of a psychiatric or neurological disorder; complications (e.g., preeclampsia) during pregnancy; and reports of illicit drug or alcohol use during pregnancy.

### Intervention and data collection

All parents of eligible participants were informed about the study and provided written informed consent prior to participation. They were informed that the study was completely voluntary, and withdrawal from the study was available at any time without any negative repercussions. Allocation to the breastmilk-fed group was based on whether mothers had adequate breast milk for their preterm infants. When the participants were enrolled in the study, according to their feeding type, the participants were divided into one of two groups: breastmilk-fed group and formula-fed group. The breastmilk-fed group was gavage-fed breast milk for a minimum of 90 days, and the formula-fed group received a mixture of breast milk and formula (the volume of formula milk comprised 80% more than that of breast milk).

At approximately term-equivalent age, an MR scan was performed. All infants were sedated using oral administration of 10% chloral hydrate (20–30 mg/Kg) before MRI examination. An experienced neonatologist who was specially trained in MRI safety was in attendance throughout the imaging process. Data with head motions of more than 3.0 mm of maximum displacement in the x, y, or z direction or 3.0° in any angular direction were discarded (see data analysis). Raw image data were collected and exported to a workstation with MATLAB software for VBM and ALFF analysis. The investigator who analyzed the data was blinded to the intervention. A self-designed questionnaire was used to obtain maternal and neonatal demographic data. Maternal data (including age, height, weight at birth) and neonatal data (including gestational age, gender, birth weight, mode of delivery, Apgar scores at 1 and 5 min, birth weight, and head circumference at birth) were recorded.

### Ethical considerations

The study protocol was approved by the Ethics Committee of the Women’s Hospital, School of Medicine, Zhejiang University. The parents of all infants eligible to participate in the study provided their informed written permission for inclusion of their infants in the study. All data were registered on the Chinese Ethics Committee of Registering Clinical Trials (ChiCTR-OON-17010460).

### MRI scan acquisition

All MR examinations were performed on a 1.5 T MR Trio scanner (Siemens Medical Solutions, Erlangen, Germany). Foam padding were used to minimize head movement during the scans. Functional images were obtained using an echo-planar imaging sequence with the following parameters: 24 axial slices, repetition time (TR) = 2000 ms, echo time (TE) = 30 ms, flip angle = 90°, field of view (FOV) = 210 × 210 mm^2^, thickness/gap = 5.0/0 mm, and in-plane resolution = 64 ×64. The resting-state between sessions lasted for six minutes. The parents were instructed to hold and comfort their babies when they cried. In addition, a T1-weighted sagittal three-dimensional magnetization-prepared rapid gradient echo sequence was acquired with the following parameters: 144 slices, TR = 2300 ms, TE = 3.39 ms, slice thickness = 1 mm, flip angle = 7°, inversion time = 1100 ms, FOV = 200 × 256 mm^2^, and in-plane resolution = 200 × 256.

### Data processing and analysis

fMRI data processing and analysis were performed using SPM8 [27]. The first 10 volumes of each participant were discarded. All images were time-shifted so that the slices were aligned temporally. Images were then realigned, and all the participants moved no more than 3 mm in translational or 3° in rotational dimensions. Images were then co-registered with the anatomical images, which were segmented into gray matter and white matter. Anatomical images were conducted with diffeomorphic anatomical registration exponentiated lie algebra (DARTEL) [28]. First, a sample-specific template was generated from the T1-weighted images. Second, individual anatomical images were nonlinearly normalized to the standard neonatal template (UNC infant template) [29] followed by linear registration to the MNI (Montreal Neurological Institute) template. Images were smoothed using a Gaussian filter with a full width at half maximum of 6 mm. Finally, nuisance signals, including the 24 head-motion parameters, cerebrospinal fluid signals, and white-matter signals were regressed from the MRI data. Further processing, including removal of linear trend and temporal bandpass filtering (0.01–0.08 Hz) and ALFF was performed using the Resting-State fMRI Data Analysis Toolkit [30].

### VBM analysis

Magnetization-prepared rapid gradient-echo (MP-RAGE) structural scans were analyzed using SPM8. SPM8 was set up for the optimized VBM protocol with no additional steps required. Optimization was performed in the segmentation process, where the gray and white matter and cerebrospinal fluid (CSF) were segmented and three new images were created. During segmentation, SPM8 warps a set of tissue probability maps, overlaying the images to segment the gray and white matter and CSF with improved accuracy. In total, images underwent the entire VBM process, which included normalization, segmentation, and smoothing. Group-level statistical analyses were performed on the modulated gray matter images. The statistical analysis was performed with the same effects as the fMRI analysis.

### Statistics

Continuous variables of demographic characteristics, are presented as the mean ± standard deviation. Data from the two groups are compared using ANCOVA on infants’ weight and head circumference. Categorical variables are presented as the number of participants (percentage). Data were analyzed with a chi-square test for multinomial distributions and with Fisher’s exact test (both two-tailed). To account for the influence of confounding factors, all variables are done adjustments using regression models with related factors. Two-sided p-values less than 0.05 were regarded as significant. Statistical analyses were performed with SPSS 20.0 software (SPSS Inc., Chicago, IL).

Group-level statistical analyses were performed using a non-random-effects model in SPM8. Following image alignment, non-brain signal removal, and correction, a two-sample t-test was conducted on the individual mean ALFF (mALFF) maps of the two groups with small volume correction for one-sample results. Multiple comparisons correction for the results was performed using the Monte Carlo simulation (see program AlphaSim by B.D. Ward, http://afni.nimh.nih.gov/pub/dist/doc/manual/AlphaSim.pdf). Significant between-group differences met the criteria of uncorrected p < 0.01 for voxel level and cluster size > 40 voxels, corresponding to a corrected p < 0.05. To examine the altered ALFF difference, one-sample t-tests were conducted on the individual mALFF of between-group peak voxels in the two groups, with a significant criterion of p < 0.05 in the SPSS 20.0 software package.

Finally, we explored the dissociable anomaly of the ALFF pattern between the two groups in the whole brain with the criterion of corrected p < 0.05 for voxel level and cluster size > 228 voxels. The alpha for all significant results was two-tailed unless otherwise specified.

## Results

### Participant demographics

**Fig 1:**
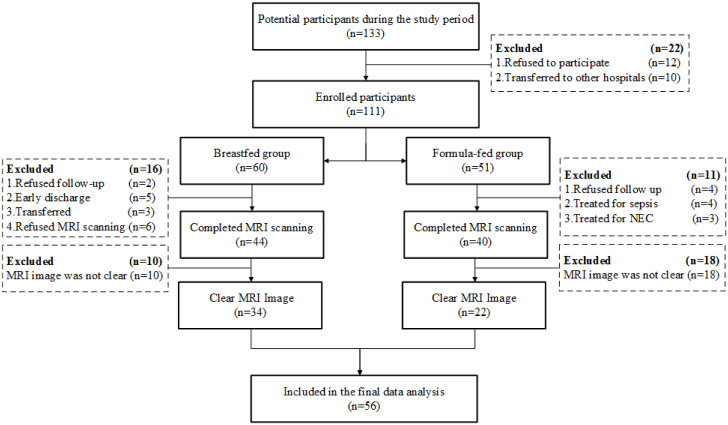
Flow chart showing the recruitment details of the study.

According to the inclusion criteria, 133 infants were enrolled in the study. During the study period, 55 preterm infants did not complete the entire study because of parent refusal to participate, loss to follow-up, or hospital transfer. Fifty-six participants were included and completed the analyses (Fig 1).

Thirty-four breastmilk-fed infants (mean gestational age 32.21±0.85 weeks) and 22 formula-fed infants (mean gestational age 31.95±1.29 weeks) were included in the study. Between the two groups, no significant difference was found in the maternal and neonatal variables (p >0.05) (Table 1). All variables were retained in the regression equation in which we assessed the contributions of potential confounders in the relationship between MRI results and type of infant feeding. The effect of the variables (such as mode of delivery, weight after discharge, head circumferences after discharge, infants’ gender) on the relationship between MRI results and feeding was not identified. The reasons for preterm birth were also not different between the groups.

**Table 1:** Maternal & neonatal demographics.

Variables (Mean±SD)	Breastmilk-fed	Formula-fed	P Value
	(n = 34)	(n = 22)	
**Gestational Age (weeks)**	32.21±0.85	31.95±1.29	0.406
**Mother’s Age (years)**	30.06±4.34	31.41±4.98	0.742
**Mother’s Height(cm)**	160.85±4.41	158.68±4.83	0.188
**Mother’s Weight at Birth(kg)**	67.30±10.06	68.30±9.94	0.713
**Timing of MRI (weeks)**	39.97±1.24	40.00±1.27	0.849
**Percentage of Breast Milk(%)**	91.40±7.84	25.80±21.8	<0.001
**Infant Gender**			
Male, n(%)	19 (55.9)	14 (63.6)	0.715
**Twins, n(%)**	8 (23.5)	9 (40.9)	0.167
**Mode of Delivery**			
Vaginal Delivery, n(%)	9 (26.5)	2 (9.1)	0.07
Cesarean Section, n(%)	25 (73.5)	20 (90.9)	
**Apgar (1 min)**	9.24±1.30	8.95±1.36	0.440
**Apgar (5 min)**	9.88±0.32	9.50±0.67	0.312
**Birth Weight(kg)**	1839.8±345.9	1800.1±364.7	0.683
**Weight After Discharge(kg)**	2367.7±350.6	2437.8±427.9	0.493
**Head Circumference at Birth(cm)**	30.27±1.65	29.80±1.72	0.287
**Head Circumference After Discharge(cm)**	31.88±1.33	32.47±1.26	0.086
**Duration in Hospital(days)**	25.56±12.02	26.14±1.59	0.901
**Reasons for Preterm Delivery**			
Premature Rupture of Membrane, n(%)	13 (38.2)	4 (28.2)	0.225
Placental Abruption, n(%)	0 (0)	2 (9.1)	
Gestational Hypertension, n(%)	4 (11.8)	1 (4.5)	
Multiple Birth, n(%)	6 (17.6)	6 (27.3)	
Other*, n(%)	11 (32.4)	9 (40.9)	

*Other reasons include cicatricial uterine pregnancy and intrahepatic cholestasis of pregnancy.

### ALFF analyses

**Fig 2:**
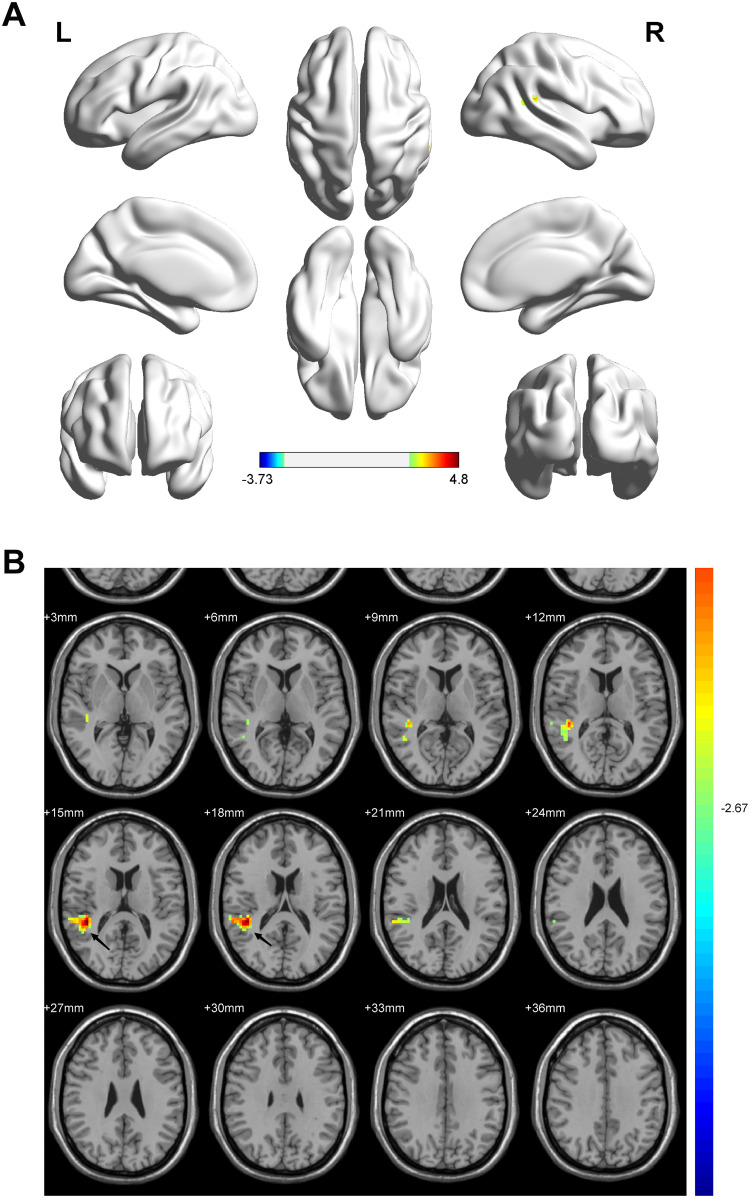
Comparison of regional brain activation measures by ALFF. The results are shown in a 3D view in A and a 2D view in B. Average activation maps for the fMRI under analgesia for breastmilk-fed and formula-fed infants. The threshold was set at p < 0.01 after the Bonferroni correction given a cluster size ≥40 voxels. All images are displayed in the radiologic convention (left/right flipped). Breastmilk-fed infants had more extensive activation in the right temporal lobe (black arrow). The regions are highlighted in yellow/green (breastmilk-fed>formula-fed).

The average fMRI activation map (p <0.01, AlphaSim-corrected cluster size >40 voxels) showed widespread activation in the right superior temporal gyrus (BA 41) for breastmilk-fed preterm infants, whereas the formula-fed infants had fewer activation regions. The total activation cluster size in the involved brain area showed significantly more activation for breastmilk-fed infants than for formula-fed infants. (Table 2 and Fig 2).

**Table 2:** Brain areas with significant ALFF differences between the breastmilk-fed group and the formula-fed group.

Brain Regions			MNI (x, y, z)			Cluster Size	BA	T Value
Hemispheres	Lobes	Regions	x	y	z			
Right Cerebellum	Temporal Lobe	Superior Temporal Gyrus	42	-39	15	125	41	4.7974

### VBM analyses

**Fig 3:**
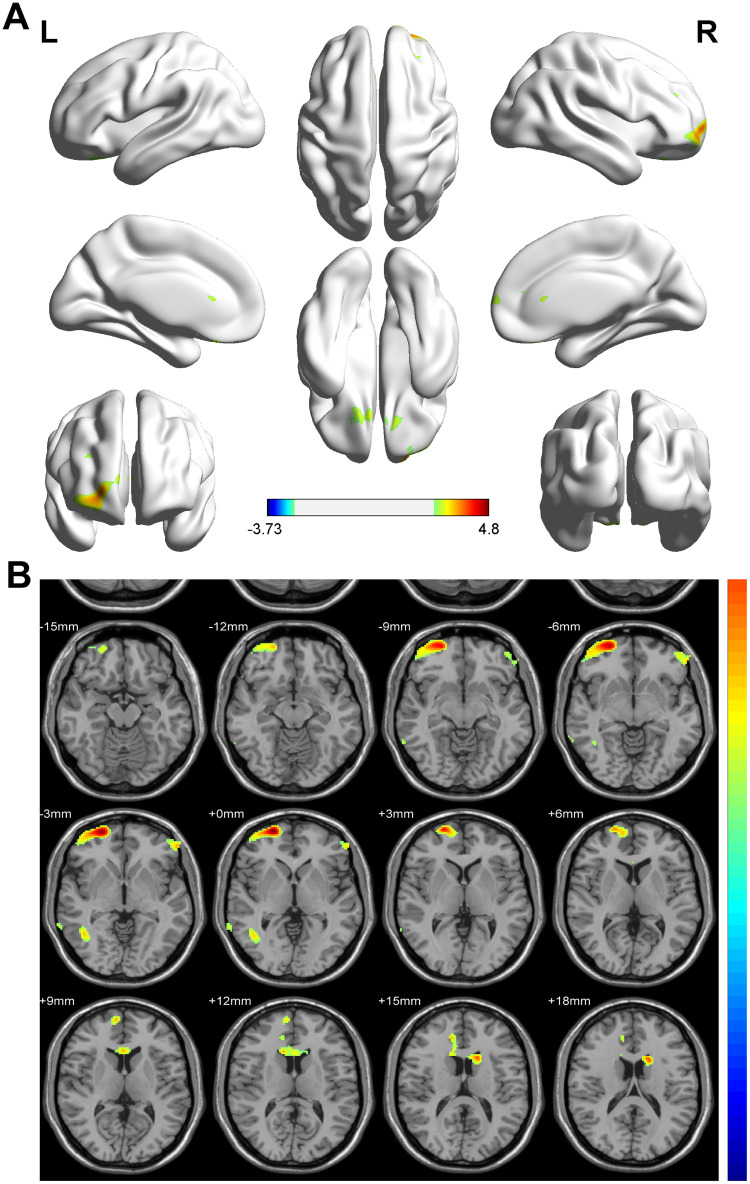
Comparison of regional gray matter volume measures by VBM. The results are shown in a 3D view in A and a 2D view in B. The threshold was set at *p*< 0.01 after the Bonferroni correction given a cluster size ≥40 voxels. Multiple regions had higher gray matter volumes in breastmilk-fed infants than in formula-fed infants. The regions are highlighted in yellow (breastmilk-fed> formula-fed). All images are displayed according to the radiologic convention (left/right flipped).

Compared with the formula-fed group, the breastmilk-fed group showed extensive areas of increased gray matter volumes involving the bilateral frontal lobe (BA11, BA46), left caudate nucleus, and right temporal lobe (BA37) at a statistical threshold of p <0.01 (AlphaSim corrected) with a cluster size of >40 voxels. However, there were no regions with low gray matter volume. The details of this analysis are shown in Table 3 and Fig 3.

**Table 3:** Brain areas of significant VBM difference between the breastmilk-fed group and formula-fed group.

Brain Regions			MNI (x, y, z)			Cluster Size	BA	T Value
Hemispheres	Lobes	Regions	x	y	z			
Left Cerebellum	Frontal Lobe	Middle Frontal Gyrus	-56	44	-2	153	46	3.2073
Left Cerebellum	Frontal Lobe	Inferior Frontal Gyrus	-8	28	-24	393	11	3.1965
Left Cerebellum	Caudate Nucleus	Caudate_L	-16	14	18	315	0	3.3998
Right Cerebellum	Frontal Lobe	Superior Frontal Gyrus	22	62	0	953	11	3.9382
Right Cerebellum	Frontal Lobe	Superior Frontal Gyrus	26	42	26	133	46	3.1784
Right Cerebellum	Temporal Lobe	Middle Temporal Gyrus	44	-58	-2	122	37	3.1082
Right Cerebellum	Temporal Lobe	Inferior Temporal Gyrus	64	-54	-10	49	37	2.8493

## Discussion

Although most studies on pediatric nutrition historically have emphasized the relationship between nutrition and growth rates or gastrointestinal maturation, efforts have increasingly focused on how nutrition influences brain development [31]. Optimal nutrition early in life is critical to ensure proper structural and functional development of the infant’s central nervous system [15]. The provision of human milk is considered the gold standard in neonatal nutrition [32], and is also universally recognized as the optimal feeding choice for preterm infants. Human milk contains a variety of critical constituents (e.g., DHA, cholesterol, LC-PUFAs) that could benefit the less-developed brain of preterm infants and promote optimal neurodevelopment [13]. Wang et al.’s study [33] showed that high brain ganglioside glycoprotein sialic acid concentrations in breast milk are related to increased synaptogenesis and differences in neurodevelopment. Their study also indicated that breastmilk-fed infants had a significantly higher percentage of DHA and total n-3 fatty acids in brain frontal cortex gangliosides relative to formula-fed infants. Additionally, breast milk has a complex and variable composition, which differs over time and between women. The differences in the fatty acid profiles of human milk and infant formula might make a difference in subsequent brain structure and function. However, there are currently few MRI studies to indicate that breastfeeding may improve early brain development in preterm infants [16, 34]. Our quasi-experimental MRI study was designed to explore the relationship between breastfeeding and early brain structural and functional development of preterm infants around term-equivalent age.

Despite no significant difference in maternal and neonatal variables between the two groups, the results of VBM analysis showed multiple brain regions with significantly higher gray matter volume in breastmilk-fed infants compared with formula-fed infants. These findings are consistent with Herba et al.’s study [35], wherein breastmilk-fed infants showed higher regional gray matter volume than formula-fed infants. Our study showed extensive areas of increased gray matter volumes involving specifically the bilateral frontal lobe, left caudate nucleus and right temporal lobe in breastmilk-fed infants compared with formula-fed infants.

Aoyama et al.’s study [36] showed that the odor of maternal breast milk induced a significantly greater increase in the oxygenated blood flow to the orbitofrontal region than the odor of formula milk did. This finding suggested that the odor of maternal breast milk may stimulate the development of the frontal lobe. The frontal lobes play an important role in many processes, such as motor control, voiding, cognition, attention, memory, language, and neuropsychiatric function [37]. Patra et al.’s study [38] of very low birth weight infants also indicated a dose-dependent relationship between increased breast milk intake in the NICU and improved cognitive outcome of infants at a corrected age of 20 months. Meanwhile, recent studies have showed that early nutrition is associated with improved white matter maturation. Coviello et al.’s study [16] investigated the effect of nutrition and growth during the first four weeks after birth on cerebral volumes and white matter maturation at term equivalent age (TEA) and on neurodevelopmental outcome at two years’ corrected age, in preterm infants, and found a positive relationship between nutrition, white matter maturation at TEA, and neurodevelopment in infancy. Ottolini KM et al.’s small exploratory study [34] of very low birth weight preterm infants demonstrated significantly greater regional brain volumes and white matter microstructural organization by term-equivalent age. Another MRI study on adolescents(mean age15 year 9 month) by Isaacs et al. [39] supported the hypothesis that one or more constituents of breastmilk promote brain development at a structural level. Their study also showed a dose–response correlation between early breast milk intake and later IQ and whole-brain volume in adolescence. Recently Sato et al.’s randomized clinical trial [18] of children at five years old born very low birth weight showed the long-term impacts of early nutrition on white matter microstructure, which in turn is related to cognitive outcomes.

Furthermore, experimental studies in animals have shown that brain structure and function are permanently affected by early nutrition. Liu et al.’s study [40] on neonatal piglets indicated that the rich source of phospholipids and gangliosides in breast milk could improve spatial learning and affect brain growth and composition. Our findings, where breast milk, which contains a greater source of important phospholipids and gangliosides, attributed to increased gray matter in regions in the brain, may contribute to a positive effect on later cognitive, behavior, memory and language development, however further studies will be required to confirm this. Moreover, early diet effects on gray matter are prominent in deep regions during infancy [41]. We also found that the size of the left caudate nucleus (part of the brain associated with memory and language) was notably larger in breastmilk-fed infants than in formula-fed infants. Specifically, communication skills are thought to be controlled mostly by the left caudate and the thalamus. A study by Belfort et al. [42] found that predominant breast milk feeding in the first 28 days of life was associated with increased deep nuclear gray matter volume at term-equivalent age, and the same study also indicated that children who received more breast milk in their first month of life also scored significantly higher on tests of IQ, working memory, mathematics, and motor skills at seven years of age.

Our VBM analysis showed that breastmilk-fed infants had advanced brain structural development in the right middle and inferior temporal gyrus. Our results differ from those of Ou et al. [43], who reported a significant gray matter volume difference in the inferior temporal lobe of the left brain between breastmilk-fed and formula-fed children at eight years of age. Left hemisphere development is more protracted than the right hemisphere. A study by Chiron et al. [44] supports the hypothesis that the right hemisphere develops its functions earlier than the left hemisphere. In their study, the blood flow showed a right hemispheric predominance mainly due to the activity in the posterior associative area between one and three years of age. Brain asymmetry shifts to the left after three years. The subsequent time course of changes appears to follow the emergence of functions localized initially in the right hemisphere, but later in the left hemisphere. These findings might explain why our results differed from Ou et al.’s study [43] in children who were eight years old.

ALFF indirectly reflects spontaneous neural activity and indicates functional changes in brain activity [45, 46]. Our fMRI results indicated that breastmilk-fed infants may have better functional development than formula-fed infants in early life. Most importantly, we found a higher blood oxygen level–dependent (BOLD) response in the right superior temporal gyrus in breastmilk-fed infants than in formula-fed infants. The temporal lobe is associated with auditory, visual, memory, and language functions. Ou et al.’s study showed that compared with formula-fed children, breastmilk-fed children had significantly more activation in the bilateral temporal lobe for the perception task and in the left temporal lobe for the language task. In Dhillon et al.’s study [47] transient evoked otoacoustic emission responses in the breastmilk-fed group were significantly larger over the low to middle frequencies, which suggested that breast milk feeding might improve middle ear function. Khedr et al.’s study [48] demonstrated that visual evoked potential, brainstem auditory evoked potential, and somatosensory evoked potential are more mature in breastmilk-fed infants than in formula-fed infants at one year of age, suggesting breast milk feeding helps earlier development and maturation of some aspects of the nervous system than milk formulas. The short term effect of breastfeeding on auditory perception needs further research. Cheatham et al.’s observational study [49] of the effects of nutrition on recognition memory revealed that high choline and DHA in human milk were associated with superior recognition memory in infants at six months of age. Moreover, the greater brain activation in the right temporal lobe observed in breastmilk-fed infants is consistent with our VBM findings, in which higher volumes of regional gray matter were detected. Pivik et al.’s study [50] of infants at three to six months old indicated that breastfeeding improves the development and processing of language stimuli during the first six months of life, suggesting breast milk might help to establish the basis for later language acquisition. Additionally, Belfort et al.’s prospective cohort study [51] found that breastfeeding was associated with better language development at age three years and higher intelligence at age seven years when compared with formula-fed. To summarize, breastfeeding appears to impact both short- and longer-term brain development.

### Limitations

The primary limitation of this study was the small sample size of preterm infants and the observational methodology, however our sample size was typical of a MRI study in infants. Another limitation was the duration of our study; we only investigated the short-term impact of breast milk in preterm infants. Also, we didn’t include gestational age at birth as covariate factors for the analysis. Future studies with larger sample sizes, follow-up of cohorts at early childhood and inclusion of more covariate factors may be beneficial to better indicate neuroimaging correlates of breast milk feeding effects on early brain development in preterm infants.

## Conclusions

Our findings showed that the brain structure and function in breastmilk-fed preterm infants were predominantly different to that of formula-fed infants around term-equivalent age with greater regional gray matter volume in the bilateral frontal lobe, right temporal lobe, and left caudate nucleus, and greater brain activation in the right temporal lobe in breastmilk-fed infants than in formula-fed infants. Further research in needed to better understand the relationship between breast milk intake and brain structure and function in the preterm infant population.

## Supporting information

S1 Checklist STROBE statement—Checklist of items that should be included in reports of *observational studies*. (DOCX)
